# Lost in Translation – Ambiguities in the Collective Terms Describing Suicide, Suicide Attempt, Suicidal Thoughts, and Self-Harm in the Research Literature

**DOI:** 10.1027/0227-5910/a001044

**Published:** 2026-02-13

**Authors:** Amelia Hughes, Alexandra Pitman

**Affiliations:** ^1^ UCL Division of Psychiatry, University College London, London, UK; ^2^ Department of Public Health and Primary Care, University of Cambridge, Cambridge, UK; ^3^ North London NHS Foundation Trust, London, UK

It has long been proposed that the research community requires an international nomenclature and classification system to describe the range of thoughts, communications, and behaviors relating to self-harm and suicide (Silverman & De Leo, 2016). The International Association for Suicide Prevention (IASP) has since convened a taskforce to establish a nomenclature, for the absence of a unified language has led to the use of numerous (and occasionally pejorative) terms to describe aspects of the same phenomenon, hampering a shared understanding of this key public health problem. Debates in this field include whether suicide definitions should be limited to self-inflicted acts (Marušič, 2004), thereby excluding euthanasia (Riisfeldt, 2023) or suicide-by-cop (Patton & Fremouw, 2016), and whether self-harm definitions should reference apparent motivation (Kapur et al., 2013). For researchers, such inconsistencies undermine the comparability of findings between empirical studies, and over time, weaken the validity and power of meta-analytic conclusions. Consequently, we continue to under-ascertain the burden of self-harm, suicidal ideation, and suicide; mischaracterize features of the groups at greatest risk; and miss opportunities for prevention and distress reduction (Silverman et al., 2007a; Silverman et al., 2007b). Meanwhile, amid steady growth in the annual volume of biomedical publications, the language of suicide-related terms continues to evolve.

There is striking variation in the collective terms referring to these phenomena– how authors set the bounds of what they include and exclude when they refer variously to suicidality, suicidal behavior, suicidal thoughts and behaviors (STBs), suicide-related outcomes, self-injurious behaviors, and self-injurious thoughts and behaviors (SITBs). Some encompass nonsuicidal self-injury (NSSI), a construct that is understood to serve an emotion regulation function (Klonsky et al., 2014). Recognizing debates over whether we conceptualize self-harm as suicidal/nonsuicidal or on a continuum (Moran et al., 2024), consensus over the association of NSSI with suicide (Klonsky et al., 2014) affirms its place within this research field for its contribution to our understanding of suicide risk factors. But the key problem the community contends with is the confusion that arises when terminology is inconsistent– using identical or different terms to refer to the same phenomena (see Table 1).

**Table 1:** Collective terms used to describe suicide and self-harm and their varying definitions

Collective term	Narrowest definition	Other definitions within this spectrum	Broadest definition
Suicidality	Tighter definitions refer to suicidal thoughts and attempts, as distinct from any nonsuicidal self-harm (McManus et al., 2022).	Originally defined as the “strength of suicidal inclinations” (Barraclough & Shepherd, 1994, p. 121). Now primarily used to encompass suicidal ideation, suicidal planning, and suicide attempt (McLaughlin et al., 2012; McManus et al., 2022; Ross et al., 2024; Wardle & McManus, 2021).	Some studies include suicide in their definition (Angelakis et al., 2019; Han et al., 2021; Meyer et al., 2010).
			Broadest definitions incorporate self-harm, suicidal thoughts, suicidal plans, and suicide (Marušič, 2004; McCloud et al., 2004).
Suicidal behavior	Defined narrowly on the WHO diagnostic classification system (International Classification of Diseases; ICD) as “concrete actions, such as buying a gun or stockpiling medication, that are taken in preparation for fulfilling a wish to end one's life but that do not constitute an actual suicide attempt” (WHO, 2022, Code: MB23.S)	Defined in the Columbia Suicide Severity Rating Scale (C-SSRS) as acts that are clearly separate from suicidal thoughts (Posner et al., 2011).	Generally used to describe both suicide attempt and suicide (Knipe et al., 2019; Ross et al, 2024; Short et al., 2024), sometimes further subclassifying these as fatal or nonfatal suicidal behavior (Knipe et al., 2019).
		The American Psychiatric Association’s diagnostic classification system (Diagnostic and Statistical manual of Mental Disorders; DSM) proposed suicidal behavior disorder as applying to anyone who has made a suicide attempt in the last two years, not counting suicidal ideation, preparatory acts or NSSI (Oliogu & Ruocco, 2024), and as it is presented as a DSM diagnostic code rather than one employed for deaths (so-called Z codes), this implies the exclusion of suicide.	
		Some survey studies define it as suicidal ideation and suicide attempts (Kaess et al., 2014), others use it to refer only to suicide attempt (Canal-Rivero et al., 2025), which may be influenced by data sources where a study relies on living subjects (Canal-Rivero et al., 2025; Posner et al., 2011).	
		Some articles have used it to both include and exclude suicidal ideation (Turecki & Brent, 2016).	
Suicidal thoughts and behaviors (STBs)	Strict definitions cover suicidal thoughts, plans, and attempts (Mortier et al., 2018).	Sometimes used interchangeably with the term suicidality (Kazan et al, 2016).	Sometimes expanded to include suicide (DiBlasi et al 2021; Kazan et al, 2016).
Suicide-related outcomes	Narrower definitions are confined to ideation and attempt only (Bruno et al., 2023).	Sometimes used to refer to suicidal ideation, attempts, and suicide deaths (Katz et al., 2022; Mulligan et al., 2024; Na et al., 2025).	Broader definitions expand to include death wish, suicidal ideation, suicidal planning, self-harm, suicide attempt, and suicide death (Torino et al., 2025).
Self-injurious behaviors (SIBs)	Strictly used as a synonym for NSSI and clearly distinguished from suicide attempt (Kaess et al., 2014; Muehlenkamp, 2005; Muehlenkamp & Gutierrez, 2004).	N/A	Sometimes used as an umbrella term for both NSSI and suicide attempt (Klonsky et al., 2014), but not generally used to include suicide.
Self-injurious thoughts and behaviors (SITBs)	N/A	N/A	Refers to any thought or act of self-injury of an individual, irrespective of suicidal intent, including suicide (Fox et al., 2018), except where methods preclude this (Janssens et al., 2024).
Self-destructive behaviors	N/A	Commonly used to refer to self-injurious behaviors, suicidal ideation, and suicide attempts, but not suicide (Kaess et al., 2014).	Sometimes used as an extended self-harm definition to include hazardous sports, excessive exercise, stopping indicated medication, excess alcohol, restrictive eating, and body modifications (Moran et al., 2024; Skegg, 2005), but not generally used to refer to suicide.
*Note*. N/A = not applicable.			

The term *suicidality* implies the state of being suicidal and was described in 1994 as a “recent invention … a noun for the strength of suicidal inclinations” (Barraclough & Shepherd, 1994, p. 121). Having gained traction as a useful shorthand, its meaning has diversified, the main contradiction centering on whether it includes suicide or not. The term *suicidal behavior*, used similarly inconsistently, implies that it relates to actions taken (Davis et al., 2015), suggesting that ideation lies outside its scope. However, usage varies perhaps the most of all collective terms, both including and excluding ideation, with clarity over its use seeming more pressing given the drive to recognize suicidal behavior as a distinct mental disorder (Sisti et al., 2020). The umbrella term *suicidal thoughts and behaviors* (STB) offers advantages in clearly distinguishing thoughts and is probably used the most consistently, except where encompassing suicide. Those using the term *suicide-related outcomes* (or suicide-related events) are not always clear whether this captures outcomes inclusive of, or additional to, suicide. Self-harm is indeed “related to” suicide, but is rarely included in this term. Definitional contradictions position the term *self-injurious behaviors* (SIB) as both the broadest and narrowest term used in the literature, while the terms *self-injurious thoughts and behaviors* (SITBs) and *self-destructive behavior*s are possibly the broadest labels of all, used relatively consistently. It seems that definitions are sometimes shaped by methodology (where a dataset lacks recording of suicide or of ideation), sometimes by restrictive word counts, but predominantly by lack of a clear lead. When definitions were debated at a 2009 consensus-building conference, experts concluded that the term suicidality was not as valid or clinically useful as the use of the specific constituent components of its various definitions in the trial literature (Meyer et al., 2010). The same appears to apply to all collective terms in current usage.

The above overview identifies SITB as the collective term used in the most consistent way by researchers, pointing toward its utility and potential candidature as a standard umbrella term, provided that its boundaries can be agreed, particularly in relation to suicide. Agreement on a common language, as achieved for other psychological constructs (Liesefeld et al., 2024), is likely to be difficult (Silverman & De Leo, 2016). The different cognitions associated with each constituent construct, along with the multiple theoretical models underpinning them, have yet to be integrated into a cohesive, unitary theory (Cha et al., 2019). Pending consensus, ambiguity is avoided if all researchers set out their definitions at first mention, clarifying their inclusion and exclusion criteria. In syntheses of the literature, clearly specifying the exact outcome(s) when discussing individual study findings also helps reduce the chance of misunderstandings (Janssens et al., 2024; Schmaal et al., 2020). Nevertheless, working with any given definition can be challenging; for example, where a systematic review is unable to separate nonsuicidal self-harm from suicide attempt because included studies lack data on intent (Knipe et al., 2019).

These definitional issues have implications for the branding and identity of its research community. The term *suicidology* was coined in 1967 to describe the interdisciplinary study of “suicidal phenomena and their prevention” (Berman et al., 2021, p. 165) and has been interpreted more widely to encompass the study of “suicidal thoughts, intentions, motivations, and self-destructive behaviors” (Silverman et al., 2006, p. 519). The prefix *suicidol*- may convey that suicidologists are interested in suicidal (but not nonsuicidal) thoughts and acts, and could alienate research subjects who do not identify as feeling suicidal. Yet many suicidologists also study NSSI for the insights it brings as a suicide risk factor and the challenges over validating intent. Even researchers whose work is focused narrowly on any one concept in this field collaborate with researchers covering related concepts, with many of the principles of intervention and prevention being common to suicidal and nonsuicidal thoughts and acts (Knipe et al., 2022).

What our research lenses all focus on is a community of individuals who struggle with self-harm yet differ in terms of what support they need. The label “suicide and self-harm researcher,” while less succinct than suicidologist, acknowledges more explicitly that not all self-harm is enacted with suicidal intent. It also accommodates an understanding of the role of nonsuicidal self-harm in reducing suicide risk for some individuals (Herzog et al., 2022). The study of all these phenomena offer insights; the more we understand the cognitions and emotions underlying such drives, the better informed we are in developing appropriate interventions, whether for suicide prevention or distress alleviation.

Only very few terminological examples described above reflect research in low- and middle-income countries (LMICs), reflecting the imbalance in empirical research internationally relative to geographic suicides (Knipe et al., 2019). The underlying meaning of suicidal thoughts and acts is likely to vary cross-culturally. Seeking universally applied clear-cut definitions and categorizations runs the risk of ignoring these differences and imposing our own interpretations. In clinical contexts, intent is clinician-inferred (Silverman et al, 2007a). In research contexts, capturing intent relies on the research measures used, yet these lack linguistic, conceptual, normative, and scale equivalence in different cultural settings (Colucci, 2006). Asking participants about a “past suicide attempt” might appear explicit to the researchers (in excluding nonsuicidal self-harm) yet elicit very different responses in different cultural settings. The development of valid measurement instruments that distinguish between thoughts and acts based on meanings and motivations in different cultures is essential for gaining the definitional consensus we need for comparability of findings.

Clarifying the meaning of intent lies at the heart of ambiguities in defining suicide and self-harm constructs, and clearly also muddies the collective terms used for capturing their various permutations. Yet inferring intent is extremely challenging, particularly in settings where the cultural validity of survey measures is in question (Andriessen, 2006) or where the cognitive and physical availability of highly lethal methods obscures whether an act was intended to be fatal (Moran et al., 2024). While having a sufficient complement of locally valid measurement instruments might help progress debates over nomenclature and classification systems, the long wait for these hampers progress toward an international consensus.

In reviewing these examples of inconsistent usage of collective terms, this Viewpoint points to SITB as a term where usage converges best and highlights the need for us to be more wary in our critical appraisal of the terms different authors employ. Most importantly, it reminds all of us to write research protocols and papers that explicitly define the bounds of our umbrella terms (and outcome measures). While investing in local efforts to improve the validity of measurement instruments, we could continue international consensus-building on terminology. Agreement on the specific components of the collective terms used throughout this literature will promote clear communication of clinical and epidemiological findings among researchers, policymakers, clinicians, and the public, conveying key messages to those who need to hear them and supporting progress toward our research, policy, clinical, and public health goals.

## Notes

**Publication Ethics:** Alexandra Pitman is an action editor of the journal. She was excluded from the peer review and editorial evaluation of the manuscript prior to acceptance. Thomas Niederkrotenthaler handled the peer review of this paper.
